# The Influence of Position of the Displaced Lesser Trochanter on Clinical Outcome of Unstable Trochanteric Femur Fractures in the Elderly

**DOI:** 10.1155/2018/5013646

**Published:** 2018-10-21

**Authors:** Qi Sun, Wei Ge, Hengda Hu, Gen Li, JieZhou Wu, Guanghua Lu, Ming Cai

**Affiliations:** Department of Orthopaedics, Shanghai Tenth People's Hospital, Tongji University, School of Medicine, Shanghai 200072, China

## Abstract

**Purpose:**

This study aimed to evaluate whether position of the displaced lesser trochanter affected clinical outcome in the treatment of unstable trochanteric fractures with intramedullary fixation.

**Patients and Methods:**

Patients with unstable trochanteric fractures and displaced lesser trochanter who received intramedullary fixation were retrospectively reviewed in this study. Based on displacement distance of the lesser trochanter and whether the lesser trochanter was reduced operatively, patients were divided into three groups: patients with the displaced lesser trochanter less than 1cm (Group A), those with the displaced lesser trochanter more than 1 cm without operative reduction (Group B), or those with operative reduction (Group C). The surgical time, reduction quality, Harris Hip Score (HHS), Visual Analog Score (VAS), and complication rate were reviewed.

**Results:**

There were 42 patients in Group A, 33 in Group B, and 36 in Group C with comparable demographic characteristics. The surgical time was significantly longer in Group C (P=0.009), compared with Groups A and B. Fracture reduction quality was comparable with over 85% good reduction among the three groups. The VAS score was significantly higher in Group B (P=0.023) without significant difference between Groups A and B. The HHS score was slightly lower in Group B, but it did not reach significant difference. The complication rate was statistically higher in Group B (p=0.043) than Groups A and C.

**Conclusion:**

The severe displaced lesser trochanter may increase postoperative complications and postoperative pain in the treatment of unstable trochanteric femur fractures. However, the displaced lesser trochanter may not affect hip function.

## 1. Introduction

Hip fractures are a leading cause of disability among the older adults. Trochanteric femur fractures are the most common type of hip fractures and affect an increasing portion of population [1–3]. There are several classifications of trochanteric fractures that mainly divide trochanteric fractures into unstable and stable fractures [4]. Compared with stable trochanteric fractures, higher complication rate and limited hip function may occur in the treatment of unstable trochanteric fractures, which are generally determined by posterolateral instability [5, 6] and comminution of the medial cortex [4, 7, 8].

The surgical treatment of unstable trochanteric fractures is largely either intramedullary or extramedullary fixation [9, 10]. With regard to biomechanical property, no statistical differences are reported between intramedullary and extramedullary fixation in the treatment of unstable trochanteric femur fractures [11]. However, intramedullary fixation is reported with several advantages over extramedullary fixation, including minimally invasiveness, less operative time, and ease of application [12–14]. In addition, approximately 70% surgeons primarily use intramedullary fixation in the treatment of trochanteric fractures and it has become the dominant choice in the United States [15]. At present, intramedullary fixation is gradually becoming the gold standard for unstable trochanteric femur fractures.

Clinically, the displaced lesser trochanter is usually not reduced when intramedullary fixation is used in the treatment of unstable trochanteric fractures. However, posteromedial cortical support including displaced lesser trochanter is important for trochanteric fracture stability [16]. Moreover, impingement of the displaced lesser trochanter on the ischium, adjacent nerve or vessel is a potential cause of hip pain [17]. Therefore, the position of the displaced lesser trochanter may affect clinical outcome in the treatment of unstable trochanteric fractures. To our knowledge, there was only one study to evaluate whether the integrity of the lesser trochanter affected the surgical outcome, and no significant difference was found [18]. However, whether the extent of displacement and operative reduction of the lesser trochanter have an influence on hip function and postoperative complications still remains controversial. To clarify this tissue, we aimed to evaluate the influence of postoperative position of the displaced lesser trochanter on hip function and postoperative complications in the treatment of unstable trochanteric femur fractures with intramedullary fixation.

## 2. Method and Materials

### 2.1. General Information

The retrospective study was approved by Institutional Ethical Committee of Shanghai Tenth People's Hospital, Tongji University School of Medicine. From 2013 to 2015, patients with unstable trochanteric fractures and displaced lesser trochanter who were treated with intramedullary fixation were reviewed in this study. Inclusion criteria included unstable trochanteric femur fractures with displaced lesser trochanter treated by proximal femoral nail (PFN), proximal femoral nail antirotation (PFNA), trochanteric fixation nail (TFN), or Gamma Nail; older than 60 years old; a minimum of 1-year follow-up. Exclusion criteria included previous hip fracture, combined subtrochanteric fractures, walking disability before injury, pathological fractures, multiple fractures, open fractures, soft-tissue infection at the fracture site, or severe cardiovascular diseases. According to displacement degree of the lesser trochanter based on preoperative CT scan and whether the displaced lesser trochanter was reduced operatively, these patients were divided into displacement of the lesser trochanter less than 1 cm (Group A); displacement of the lesser trochanter more than 1 cm without operative reduction (Group B), or with operative reduction (displacement of the lesser trochanter < 1 cm after reduction) (Group C).

### 2.2. Surgical Procedure

Surgery was performed after confirmation of the suitable general health conditions of patients. All patients were placed in supine position on a fracture table under general anesthesia. Under the control of C-arm fluoroscopy, closed reduction was performed to reconstruct a near anatomical reduction of proximal femur by holding the leg under controlled traction. In Groups A and B, intramedullary nailing was subsequently performed without additional reduction and fixation of the lesser trochanter. In Group C, intramedullary nailing was performed after achievement of satisfactory reduction of the displaced lesser trochanter by bone hook. Finally, the wound was closed after confirmation of satisfactory fracture reduction and fixation with the assistance of intraoperative fluoroscopy. All patients were allowed to bear weight as tolerated after surgery.

### 2.3. Follow-Up

During follow-up, the hip scores was reviewed by Harris Hip Score (HHS), hip pain was assessed by visual analog score (VAS), and complication rate of each group was also reviewed at the final follow-up. According to degree of varus/valgus and/or antervesion/retroversion, the extent of fracture reduction was divided into good (<5°), acceptable (5-10°), or poor (>10°). Screw position was assessed by tip apex distance (TAD) by postoperative radiograph.

**Figure 1:**
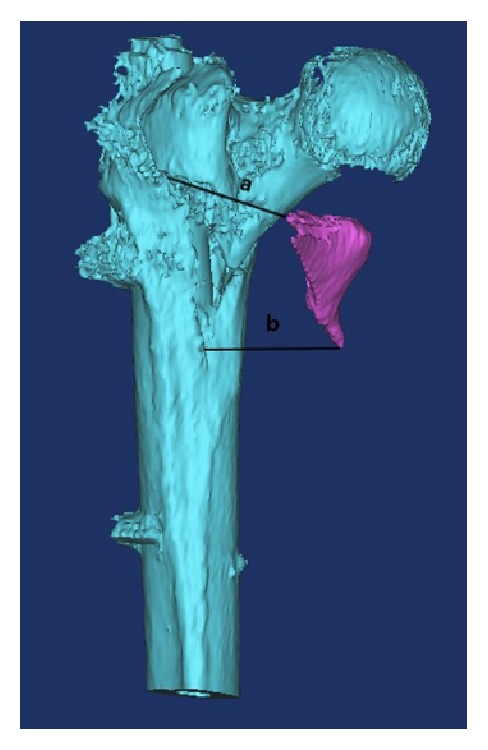
Measurement of displacement distance of the lesser trochanter.* The displacement distance of the lesser trochanter was defined as the average of distance a and distance b. Distance a was defined as the length between the highest point of fracture site and corresponding point of the lesser trochanter fragment. Correspondingly, distance b was the lowest point to corresponding point of the lesser trochanter fragment.*

The CT scan was performed immediately after surgery. Subsequently, the Mimics software (Version 14.12, Materialise N.V., Leuven, Belgium) was used for 3D reconstruction of proximal femur, based on which the measurement of displaced distance of the lesser trochanter was conducted to confirm displacement of the lesser trochanter. The displacement measurement of the lesser trochanter was performed by two experienced radiologists. The displacement distance of the lesser trochanter was defined as the average of distance a and distance b (Figure 1). Distance a was defined as the length between the highest point of fracture site and corresponding point of the lesser trochanter fragment. Correspondingly, distance b was the lowest point to corresponding point of the lesser trochanter fragment. Any patients who were not eligible in each groups were also excluded.

### 2.4. Statistical Analysis

Statistical analysis was performed with SPSS software package (version 18.0; SPSS Inc., Chicago, IL, USA) in this study. All continuous variables were summarized as mean ± standard deviations and categorical variables were summarized as frequency. One-way ANOVA and Student's t-test were applied for continuous variables, and chi-square test were used for categorical variables. A statistically significant difference was defined as p <0.05.

## 3. Result

A total of 111 patients with trochanteric fractures and displaced lesser trochanter were eligible and included in this study. There were 42 patients with an average of age of 77.7 ± 8.1 years old in Group A, 33 patients of 77.0 ± 7.9 years old in Group B, and 36 patients of 78.3 ± 8.5 years old in Group C (p=0.328). No significant differences were found in gender (p= 0.166), height (p=0.515), weight (p=0.629), and fracture side (p= 0.439) among Groups A, B, and C (Table 1). According to AO classification for trochanteric fractures, there were 39 A2 and 3 A3 in Group A, 30 A2 and 3 A3 in Group B, and 34 A2 and 2 A3 in Group C. There was no significant difference in AO classification among Groups A, B, and C (p= 0.851). With regard to fracture reduction quality, over 85% patients achieved good or acceptable fracture reduction in Groups A, B, and C. Fracture reduction quality were comparable among these groups. (Table 2.)

Compared with Group A (45.5±7.7 min) and Group B (49.5±7.5 min), the surgical time was significantly longer in Group C (61.9 ± 8.9 min). There were no statistically differences in length of hospital stay and time to bone union among Groups A, B, and C (Table 3.). However, the VAS score was 0.4 ± 0.4 (Range: 0-2) in Group A and 0.5 ± 0.3 in Group C (Range: 0-3) (p=0.023), which was statistically lower than that in Group B (1.1±0.6, Range: 0-5). Although HHS score was slightly lower in Group B, no significant difference was observed in HHS score among Groups A, B, and C.

Furthermore, the postoperative complications were 1 (2.4%) in Group A, 6 (18.2%) in Group B, and 2 (5.6%) in Group C (Table 4), which were significantly higher in Group B (p=0.043). Complication rate was comparable among Group A and Group C. Implant failure occurred in 1 patients in Group B and in 1 patients in Group C, both patients underwent revision surgery. One hip varus was observed in Group A. There were 2 patients in Group B and 1 patient in Group C with trochanteric fracture nonunion and 3 patient with thigh pain that affected normal activity in Group B. The thigh pain improved after the administration of nonsteroidal anti-inflammatory drugs (NSAIDs) and the performance of physical therapy. No other complications, such as wound infection, were observed in our study.

## 4. Discussion

To our best knowledge, this is the first study evaluating displacement of the lesser trochanter on hip function and postoperative complications in the treatment of unstable trochanteric femur fractures with intramedullary fixation. Our study showed that HHS scores were comparable among Groups A, B, and C, although HHS score was slightly lower in Group B. The VAS score was significantly lower when displacement distance of the lesser trochanter was less than 1cm or after operative reduction, compared with the displaced distance of the lesser trochanter ≥ 1cm. The complication rate was statistically higher in Group B than that in Groups A and C. Moreover, we found a longer surgical time in Group C, compared with Groups A and B. No significant differences were observed in fracture reduction quality, screw position, length of hospital stay, and time to fracture union among these three groups.

Trochanteric femur fractures were common injury in the elderly, and surgical management was the most common treatment of trochanteric fractures [15, 19]. Intramedullary fixation was a preferred treatment of unstable trochanteric femur fractures, which could result in satisfactory clinical outcomes [8, 14]. In our study, we found that patients with displaced lesser trochanter less than 1cm or with displaced lesser trochanter less than 1cm after operative reduction had a significantly better VAS score, compared with those with displaced lesser trochanter ≥ 1cm. HHS score was slightly lower in Group B, compared with Groups A and C, although it was comparable among these three groups. Therefore, severe displaced lesser trochanter may be the main reason of the differences in VAS score and HHS score. Trochanteric fractures with severe displaced lesser trochanter after operative reduction of displaced lesser trochanter can achieve similar clinical outcome to mild displaced lesser trochanter with intramedullary fixation. A biomechanical study showed that both the size of greater trochanter and lesser trochanter were important for fracture stability [20]. Giacomini et al. [21] reported that isolated displaced lesser trochanter with conservative treatment induced unsatisfactory hip function and passive movement of the hip with pain, which was also reported by Theologis et al. [22]. Furthermore, there was a potential injury to adjacent muscle, vascular, and nerve due to severe displaced lesser trochanter, which might partially explain the higher VAS score in Group B. The narrowed distance between the displaced lesser trochanter and the ischium may cause impingement with edema of intervening quadratus femoris muscle [17, 23]. Additionally, a ballet dancer with chronic hip pain was also reported because of a lesser trochanter bony avulsion [24], which might be also caused by a severe displaced lesser trochanter.

In clinical practice, trochanteric fractures with severe displaced lesser trochanter were usually complex types of fractures and caused by severe violence [25]. In our study, the surgical time was also significantly longer in Group C compared with Groups A and B. That was also in accordance with previous studies, which indicated that trochanteric fractures of A2 and A3 needed a longer operative time [18, 26, 27]. We believed that trochanteric fractures with displaced lesser trochanter treated with intramedullary fixation were much more technically demanding, especially when operative reduction of the displaced lesser trochanter was required.

The trochanteric fractures with severe displaced lesser trochanter treated with intramedullary fixation may have a higher complications compared to those with mild displaced lesser trochanter. As demonstrated in our study, patients in Group B had statistically significant higher complication rate than result of Groups A and C. However, no significant differences were observed in complication rate between Groups A and C, which suggested that complication rate decreased after displaced lesser trochanter reduced operatively in the treatment of trochanteric fractures with intramedullary fixation. Liu et al. [18] reported a comparable complication rate between patients with and without integrity of the lesser trochanter, which partially contradicted with our result. We believed that the thigh pain was analyzed and recorded as complication in our study, and no significant difference was found after thigh pain was excluded. The thigh pain in Group B might be caused by displaced lesser trochanter, which had a potential injury to adjacent muscle and nerve [17, 21, 23]. Patients with thigh pain had pain relief after administration of NSAIDs and physical therapy. The osteoporotic bone of proximal femur may lead to the two implant failure, because all three patients were female and with age over 85 years old.

The following limitations of this study should be noted. Firstly, this study was a retrospective study, the grouping was not prospectively randomized, and the follow-up time was not consistent among the three groups. Secondly, we only assessed the displacement distance of the lesser trochanter fragment; the size and displacement direction of the lesser trochanter fragment were not taken into consideration, which might have an influence on the results. However, it was hard to conduct an effective evaluation of displaced lesser trochanter fragment by considering fracture size, displacement distance and direction, and fragment rotation. Lastly, the sample size enrolled in our study was not large enough to detect large difference among the three groups and the follow-up of patients was relatively short. Large-sized prospective randomized trials with long follow-up were needed.

## 5. Conclusion

The severe displaced lesser trochanter may increase postoperative complications and postoperative pain in the treatment of unstable trochanteric femur fractures. The displaced lesser trochanter may not affect hip function. We recommend that severe displaced lesser trochanter should be reduced when unstable trochanteric femur fractures were treated with intramedullary fixation.

## Figures and Tables

**Table 1:** Baseline characteristics of patients with unstable trochanteric fractures and displaced lesser trochanter.

	Group A (n=42)	Group B (n=33)	Group C (n=36)	P
Gender				0.166
Male	17	19	13	
Female	25	14	23	
Age (years)	77.7 ± 8.1	77.0 ± 7.9	78.3 ± 8.5	0.328
Height (cm)	162.3 ±7.8	162.9 ±7.3	161.6 ±7.7	0.515
Weight (kg)	64.4 ±5.4	64.5 ±5.6	64.7 8±6.0	0.629
Fracture side				0.439
Left	19	15	21	
Right	23	18	15	
Fracture classification				0.851
A1	0	0	0	
A2	39	30	34	
A3	3	3	2	
Interval from injury to surgery (days)	5.0 ± 1.9	5.5 ± 2.8	5.4 ± 2.4	0.297
Follow-up (months)	17.2±3.1	17.6±3.9	17.9±4.2	0.306

**Table 2:** Quality of unstable trochanteric fracture reduction among Groups A, B, and C.

	Group A	Group B	Group C	p
				0.927
Good	29	21	25	
Acceptable	7	8	6	
Poor	6	4	5	

**Table 3:** Clinical outcomes of patients treated with intramedullary fixation among Group A, Group B, and Group C.

	Group A (n=42)	Group B (n=33)	Group C (n=36)	P
Surgical time (min)	45.5 ± 7.7	49.5 ± 7.5	61.9 ± 8.9	0.009
Length of hospital stay (d)	7.4 ± 3.6	7.2 ± 3.8	7.5 ± 4.0	0.672
VAS score	0.4 ± 0.4	1.1 ± 0.6	0.5 ± 0.3	0.023
TAD	19.5 ± 4.2	19.9 ± 4.0	19.2 ± 4.3	0.313
HHS score at last follow-up	85.2 ± 7.9	82.3 ± 7.6	83.7 ± 8.9	0.374
Time to fracture union (Weeks)	12.8 ± 2.3	13.3 ± 3.0	12.9 ± 2.7	0.431

Complication rate after surgery (percentage)	1 (2.4%)	6 (18.2%)	2 (5.6%)	0.043

**Table 4:** Complications of patients.

Complications	Group A	Group B	Group C
Implant failure	0	1	1
Nonunion	0	2	1
Loss of reduction	1	0	0
Severe thigh pain	0	3	0
Total	1	6	2

## Data Availability

The data used to support the findings of this study are available from the corresponding author upon request.
